# Incremental value of non-invasive myocardial work for the evaluation and prediction of coronary microvascular dysfunction in angina with no obstructive coronary artery disease

**DOI:** 10.3389/fcvm.2023.1209122

**Published:** 2023-08-14

**Authors:** Ying Li, Dandan Sun, Hanzhang Zhao, Zhiyan Qin, Wei Ji, Huihui Zhang, Ni Jiao, Bo Luan, Mingyan Ding, Fang Zhu

**Affiliations:** ^1^ Department of Cardiac Function, The People’s Hospital of Liaoning Province, Shenyang, China; ^2^ Department of Ultrasound, Affiliated Hospital of Shandong University of Traditional Chinese Medicine, Jinan, China; ^3^ Department of Cardiology, The People's Hospital of Liaoning Province, Shenyang, China

**Keywords:** stress echocardiography, angina with no obstructive coronary artery disease, coronary microvascular dysfunction, myocardial work, coronary flow velocity reserve

## Abstract

**Background:**

Evidence suggests that patients suffering from angina with no obstructive coronary artery disease (ANOCA) experience coronary microvascular dysfunction (CMD). We aimed to understand the diagnosis value of noninvasive myocardial work indices (MWIs) with left ventricular pressure-strain loop (LV PSL) by echocardiography in ANOCA patients with CMD.

**Methods:**

97 patients with ANOCA were recruited. All subjects underwent standard echocardiography with traditional ultrasound parameters, two-dimensional speckle-tracking echocardiography with global longitudinal strain (GLS), LV PSL with MWIs include global work index (GWI), global constructive work (GCW), global waste work (GWW) and global work efficiency (GWE). In addition, all enrolled cases underwent high-dose adenosine stress echocardiography (SE) with coronary flow velocity reserve (CFVR). CMD was defined as CFVR <2.0.

**Results:**

Of the 97 patients with ANOCA, 52 were placed in the CMD group and 45 in the control group. GWI and GCW were decreased significantly in the CMD group compared with the control group (*P *< 0.001 for both). GWI and GCW were moderately correlated with CFVR (*r *= 0.430, *P *<* *0.001 and *r *= 0.538, *P *<* *0.001, respectively). In the multiple logistic regression analyses, GCW was identified as the only independent echocardiography parameter associated with CMD after adjusting for age and baseline APV [OR (95%*CI*) 1.009 (1.005–1.013); *P *<* *0.001]. Moreover, the best predictor of CMD in patients with ANOCA using receiver operating characteristic (ROC) curve was GWI and GCW, with an area under the curve (AUC) of 0.800 and 0.832, sensitivity of 67.3% and 78.8%, specificity of 80.0% and 75.6%, respectively.

**Conclusion:**

MWIs with LV PSL is a new method to detect LV systolic function noninvasively in ANOCA patients with CMD.

## 1. Introduction

Approximately 40% of patients with chest pain who receive coronary angiography are diagnosed as having angina with no obstructive coronary artery disease (ANOCA) (1). Coronary microvascular dysfunction (CMD) is a condition marked by the abnormal configuration and activity of the coronary microcirculation. CMD is a well-established mediator of ANOCA (2).

CMD-triggered ischemia is comparable with obstructive epicardial coronary artery disease (CAD)-mediated ischemia. Several research revealed that, in patients suffering from CMD, there is a strong association with an enhanced risk of severe cardiovascular events such as nonfatal myocardial infarction (MI), hospitalization due to heart failure, and cardiac-related mortality (3, 4). Therefore, exploring an accurate method to evaluate the early change in left ventricular (LV) systolic function in ANOCA patients suffering from with CMD is important for treatment and management.

Coronary microvascular function cannot be assessed directly at present. However, targeting the distal of left anterior descending artery (LAD) by transthoracic doppler echocardiography makes it possible to infer coronary microvascular function indirectly. In patients not suffering from epicardial disease, a decrease of coronary flow velocity reserve (CFVR) during drug-induced stress can reflect the function of coronary microvascular (5).

Global longitudinal strain (GLS) plays an important part in the evaluation of LV systolic function. Two-dimensional speckle-tracking echocardiography (2D-STE) provides extensive quantitative information on myocardial activity in patients with CMD (6, 7). However, load dependence is a major disadvantage of GLS, and is the main factor affecting diagnostic accuracy (8).

The LV pressure-strain loop (LV PSL) with myocardial work indices (MWIs) is an accurate and noninvasive new parameter. It provides potential value for the assessment of myocardial function by accounting for the strain associated with dynamic noninvasive LV pressure. Multiple clinical works have demonstrated that MWIs have superior performance compared with GLS (9). One study demonstrated that MWIs can be used to identify early abnormalities in LV systolic function in patients with CAD. MWIs were more sensitive than GLS for detecting significant CAD in patients without abnormalities in regional wall motion (10). Another study found that, in athletes with normal cardiac function, MWIs could be employed to detect subtle subclinical changes in LV concentric remodeling after long-term intensive exercise (11). In one prospective randomized study, in patients undergoing potentially cardiotoxic chemotherapy, MWIs were superior to GLS in serial assessments of LV systolic function. Especially if there is a change in blood pressure (BP) > 20 mmHg, MWIs can provide accurate information in the surveillance for cancer treatment-related cardiac dysfunction (12). Taken together, those studies suggested that MWIs could aid detection of early changes in myocardial function. However, few reports have focused on the use of MWIs in evaluation of LV systolic function in ANOCA patients with CMD.

The present study was conducted to achieve two goals. The first goal was to assess LV systolic function via LV PSL with MWIs in patients with ANOCA who also suffering from CMD. The second goal was to provide a new method for the diagnosis of LV systolic dysfunction in ANOCA patients with CMD.

## 2. Materials and methods

### 2.1. Study design

This was a retrospective, single-center study that, between October 2019 and December 2021, evaluated 97 patients with ANOCA. Based on invasive angiography, patients with angina pectoris did not display obstruction in a coronary artery (≤50% stenosis) within the last 3 months. CFVR was obtained using high-dose adenosine stress echocardiography (SE) in all included patients. Based on CFVR values (13), patients were separated into two groups: control (*n* = 45), who had CFVR ≥2.0; CMD (*n* = 52), who had CFVR <2.0. Written informed consent was acquired from all patients before study commencement.

The inclusion criteria were: (1) age of 18–80 years; (2) symptomology of angina pectoris, with an absence of obstructive CAD (<50% diameter stenosis) within the last 3 months according to coronary angiography (14); (3) normal LV ejection fraction (LVEF) (>53%); (4) absence of heart-valve disease, cardiomyopathy, or congenital heart disease; (5) successful completion of adenosine SE; (6) satisfactory sound-transmission window.

A detailed description of the exclusion criteria (as well as the precautions for adenosine SE) has been provided previously (15–17). In addition, participants with left and right bundle branch block were excluded from evaluation. The studies involving human participants were reviewed and approved by Medical Ethics Committee of People's Hospital of Liaoning Provincial, project number (KS013).

### 2.2. Conventional echocardiography

Echocardiography was undertaken by experienced sonographer working more than five years, using an ultrasound system (Vivid E9; GE Vingmed Ultrasound, Holton, Norway) via a standard two-dimensional (2D) cardiac probe (M5S) and an Echo PAC204 post-processing workstation. Three cardiac cycles of standard 2D images were collected in DICOM format using QRS as the recording standard for offline analyses. All variables were assessed based on the recommendations of the American Society of Echocardiography (ASE) (18).

All 2D and doppler-parameter readings were conducted based on ASE guidelines in standard views. Measurements of the left atrium volume (LAV), LV end-diastolic volume (LVEDV), LV end-systolic volumes (LVESV), and LVEF were based on four- and two-chamber apical views. The LV end-diastolic dimension (LVEDD) was obtained perpendicular to the long axis of the left ventricle. Chamber recordings were standardized to the body surface area (BSA) before computing the LAV, LVEDV, LVESV, and LVEDD indices, and these parameters were finally marked as LAVI, LVEDVI, LVESVI, and LVEDDI, respectively. The mitral peak E and A velocities were obtained from the four-chamber view via pulsed wave doppler prior to E/A calculation. The mean mitral annulus and lateral wall in early diastole (e′ peak) velocities, as well as the mean mitral annulus and lateral wall in systolic (S′ peak) velocities, were acquired from the apical four-chamber view via tissue doppler prior to E/e′ calculation.

### 2.3. 2D-STE and LV PSL

Grayscale images were captured in apical two-, three-, and four-chamber views, with frame rates >50 frames/s. LV outflow pulsed doppler was utilized to record the duration of end systole. Subsequently, the operator identified three points, in particular, two on either side of the mitral valve, and a third at the LV apex. Using the 17-segment LV model, the peak GLS value from the systolic period was then recorded automatically (19).

**Figure 1:**
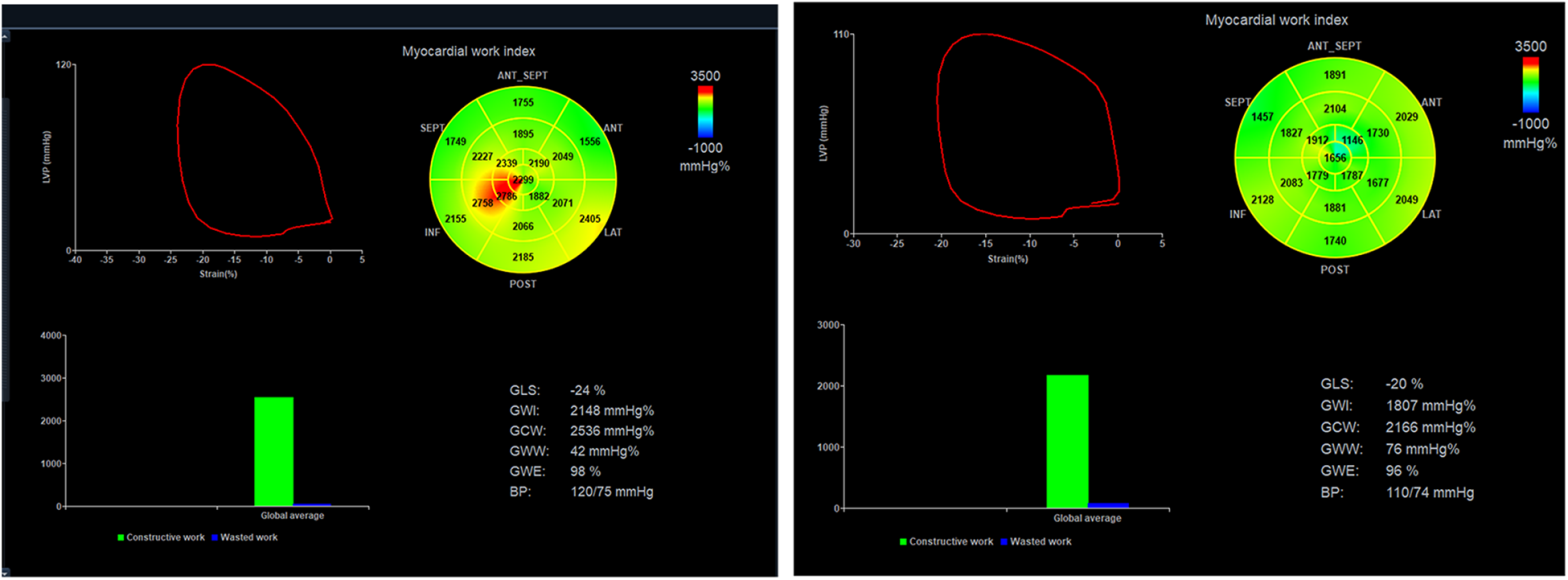
A typical case example of non-invasive myocardial work (WM) assessment. A: The pressure-strain loops (PSLs) and WM indices in the control group; B: The PSLs and WM indices in the CMD group.

Through the LV PSL, MWIs were obtained automatically as described using data from LV strain, valvular event times, and systolic BP (Figure 1). Subsequently, we derived several MWIs. GWI represented the total work within the LV PSL area, which was computed from closure and opening of the mitral valve. GCW denoted the work during shortening in systole, and addition of work during lengthening in isovolumic relaxation. GWW represented work during lengthening in systole, and additional work required during shortening in isovolumetric relaxation. Moreover, GWE was computed as the sum of constructive work in all LV segments, divided by the sum of constructive and wasted work in all LV segments. GWI, GCW, and GWW are presented as mmHg %. GWE is presented as a percentage. Normal reference values were employed for preserved-value and impaired-value definitions of MWIs (20).

### 2.4. Examination of adenosine stress

The distal LAD was acquired via modification of the two-chamber view in color doppler mode before introduction of a sample volume (width = 1.5 mm) on the color signal. Displaying the anterior interventricular sulcus in the apical region, under the guidance of color doppler blood flow, the LAD branch shows small diastolic blood flow. The probe angle needs to be carefully adjusted to minimize the angle between blood flow sound beams and record the LAD branch blood flow spectrum. The stable frequency spectrum of the LAD was obtained while recording the baseline average peak velocity (APV). Under monitoring by electrocardiography and BP, adenosine was administered for 6 min (140 μg·kg^−1^·min^−1^) using a syringe pump. The BP and heart rate were documented at 1-min intervals during intravenous pumping until all of the drug had been administered. The hyperemic peak APV of the LAD was recorded during the entire infusion. CFVR was described as the ratio of the hyperemic peak: baseline APV.

### 2.5. Intra- and inter-observer variabilities

Thirty patients were chosen arbitrarily. GLS and MWIs (GWI, GCW, GWW, GWE) were measured by two experienced observers who were not provided with the clinical data or study data. The same individuals were also assessed at different time points to ascertain intra-observer variability.

### 2.6. Statistical analyses

SPSS 25.0 (IBM, Armonk, NY, USA) was employed for all data analyses. Categorical statistics are provided as numbers and percentages. Results with a normal distribution were confirmed via the Shapiro-Wilk test. Based on the data distribution, normal data are provided as mean ± standard deviation, and non-normal data as the median (P25, P75). Analyses of data with a normal distribution were undertaken using the independent *t*-test. Analyses of data with a non-normal distribution were carried out using the Mann-Whitney test. Analyses of categorical data were based on the chi-square test. Spearman's correlation coefficient was used to test correlations of MWIs (including GWI and GCW) with CFVR.

We used univariate logistic regression analysis to identify the factors that affect CMD. In order to rule out multicollinearity issues, the independent variables included are age, baseline APV, GWI, GCW. All variables with *P* < 0.05 at univariable logistic analysis were considered for multivariate logistic regression analysis to determine the independent factor that affects CMD. Analyses of receiver operating characteristic (ROC) curves were done to evaluate the suitable threshold along with sensitivity and specificity values for MWIs and GLS in the detecting of CMD. *P *< 0.05 (two-sided) was considered significant.

## 3. Results

### 3.1. Patient demographics

One hundred and two patients met our strict criteria for inclusion and were evaluated. Five patients were eliminated from the analyses: three patients had data with poor image quality for analyses of GLS and MWIs; two patients had an incomplete adenosine stress test owing to chest pain and physical discomfort. Ultimately, we recruited 97 patients with ANOCA for evaluation and separated them into a CMD group and control group.

We observed no discernible differences in the height, weight, body mass index, BSA, tobacco-smoking history, or disease history (e.g., diabetes mellitus and hyperlipidemia) between patients in the CMD group and control group (Table 1). The CMD group was slightly older, and participated in more treatments using calcium-channel blockers than their control-group counterparts.

**Table 1:** Clinical characteristics and echocardiographic parameters of the whole study and their comparison between CMD group and control group.

Variables	All (*n* = 97)	CMD group CFVR < 2 (*n* = 52)	Control group CFVR ≥ 2 (*n* = 45)	*P*-value
Age, (years), median (P25, P75)	55.61 ± 12.26	59.44 ± 10.29	51.18 ± 12.95	**0** **.** **001**
Male, *n* (%)	35 (36.08)	18 (34.62)	17 (37.78)	0.746
Height, (cm), median (P25, P75)	165.29 ± 7.60	164.94 ± 8.30	165.69 ± 6.77	0.674
Weight, (Kg), mean ± SD	67.68 ± 11.79	67.69 ± 11.33	67.67 ± 12.44	0.992
BMI, (kg/m^2^), median (P25, P75)	24.71 ± 3.75	24.87 ± 3.89	24.53 ± 3.62	0.874
BSA, (m^2^), median (P25, P75)	1.72 ± 0.18	1.72 ± 0.18	1.72 ± 0.19	0.851
Systolic pressure, (mmHg), median (P25, P75)	134.24 ± 20.22	137.85 ± 21.12	130.07 ± 18.47	0.061
Diastolic pressure, (mmHg), median (P25, P75)	77.78 ± 12.16	77.52 ± 14.44	78.09 ± 8.97	0.819
Resting heart rate,(bpm), median (P25, P75)	73.80 ± 14.12	72.63 ± 16.41	75.16 ± 10.93	0.523
Smoking, *n* (%)	18 (17)	10 (19)	8 (17)	0.854
Hypertension, *n* (%)	56 (57)	34 (65)	22 (48)	0.101
Diabetes Mellitus, *n* (%)	13 (13)	8 (15)	5 (11)	0.538
Hyperlipidemia, *n* (%)	62 (63)	34 (65)	28 (62)	0.746
ACEI or ARB, *n* (%)	22 (22)	12 (23)	10 (22)	0.920
Beta Blocker, *n* (%)	28 (28)	15 (28)	13 (28)	0.996
Calcium Channel Blocker, *n* (%)	18 (18)	16 (23)	6 (13)	**0**.**041**
Loop Diuretic, *n* (%)	11 (11)	7 (13)	4 (8)	0.489
Aspirin, *n* (%)	61 (62)	35 (67)	26 (57)	0.333
LAVI, (ml/m^2^), median (P25, P75)	23.93 (22.86–25.17)	24.05 (23.05–25.28)	23.73 (22.64–24.89)	0.308
LVEDDI, (mm/ m^2^), median (P25, P75)	27.04 (24.98–29.07)	27.56 (25.09–29.40)	26.50 (24.88–28.17)	0.087
LVEDVI (ml/m^2^), median (P25, P75)	52.39 (48.05–57.39)	52.79 (48.38–57.55)	51.91 (47.17–56.88)	0.592
LVESVI, (ml/m^2^), median (P25, P75)	20.73 (18.61–23.15)	21.17 (19.12–23.34)	20.32 (17.94–22.22)	0.099
LVEF, (%), median (P25, P75)	61.00 (58.00–62.00)	60.00 (58.00–61.00)	61.00 (59.00–62.00)	0.073
Mitral peak E velocity, (cm/s), median (P25, P75)	71.00 (62.00–90.00)	71.00 (62.00–82.00)	71.00 (61.00–93.50)	0.519
Mitral peak A velocity, (cm/s), median (P25, P75)	80.00 (72.00–92.00)	80.50 (72.00–92.00)	79.00 (72.00–92.00)	0.534
E/A, median (P25, P75)	0.88 (0.71–1.23)	0.87 (0.70–1.22)	1.09 (0.72–1.26)	0.421
e′ peak, (cm/s), median (P25, P75)	10.00 (8.50–10.50)	9.00 (8.00–10.00)	10.00 (8.50–11.00)	0.084
E/ e′, median (P25, P75)	7.75 (6.71–9.20)	8.05 (6.69–9.41)	7.71 (6.71–8.68)	0.452
S′ peak, (cm/s), median (P25, P75)	8.00 (7.00–9.00)	8.00 (7.00–9.00)	8.00 (7.00–9.00)	0.351
Baseline APV, (cm/s), median (P25, P75)	20.00 (18.00–27.00)	22.50 (18.00–29.50)	19.00 (16.50–23.50)	**0**.**005**
Peak APV, (cm/s), median (P25, P75)	48.00 (37.00–60.00)	39.50 (34.00–50.00)	54.00 (44.00–65.50)	**<0**.**001**
GLS, (%), mean ± SD	−21.95 ± 1.36	−21.71 ± 1.42	−22.22 ± 1.24	0.084
GWI, (mmHg%), median (P25, P75)	2,107.00 (1,892.50, 2,217.50)	1,913.50 (1,786.75, 2,121.75)	2,203.00 (2,098.00, 2,359.50)	**<0**.**001**
GCW (mmHg%), mean ± SD	2,373.14 ± 200.29	2,265.73 ± 168.03	2,497.27 ± 159.43	**<0**.**001**
GWW (mmHg%), median (P25, P75)	80.00 (65.00, 102.50)	81.50 (65.00, 102.75)	78.00 (67.00, 102.50)	0.985
GWE, (%), median (P25, P75)	96.61 (95.85, 97.29)	96.39(95.76, 97.31)	96.82(95.96, 97.26)	0.623

CMD, coronary microvascular dysfunction; CFVR, coronary flow velocity reserve; BMI, body mass index; BSA, body surface area; ACEI, angiotensin-converting enzyme inhibitor; ARB, angiotensin receptor blocker. LAVI, left atrium volume index; LVEDDI, left ventricular end-diastolic dimension index; LVEDVI, left ventricular end-diastolic volume index; LVESVI, left ventricular end-systolic volume index; LVEF, left ventricular ejection fraction; e′, average tissue doppler velocities of the mitral annulus and lateral wall in early diastole; S′, average tissue doppler velocities of the mitral annulus and lateral wall in systolic; APV, average peak velocity; GLS, global longitudinal strain; GWI, global work index; GCW, global constructive work; GWW, global waste work; GWE global work efficiency. *P* < 0.05 is marked with bold type.

### 3.2. Analyses of echocardiography parameters

**Figure 2:**
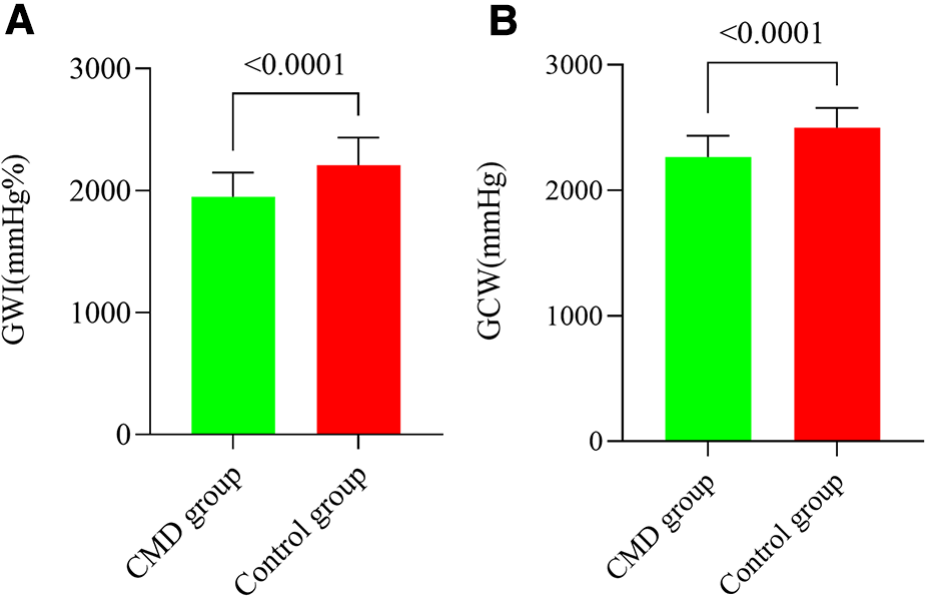
GWI and GCW among CMD patients and control counterparts. (**A**) GWI was strongly diminished among the CMD group. (**B**) GCW was strongly diminished among the CMD group. (**A**) GWI values provided as mmHg%. (**B**) GCW values provided as mmHg%.

**Figure 3:**
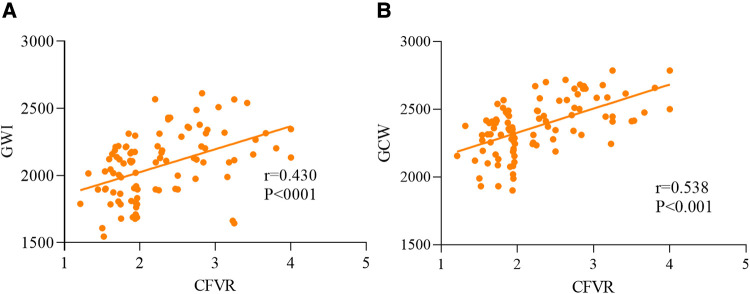
Analysis of correlation of the MW parameters GWI (**A**) and GCW (**B**) with CFVR in ANOCA patients with CMD.

**Figure 4:**
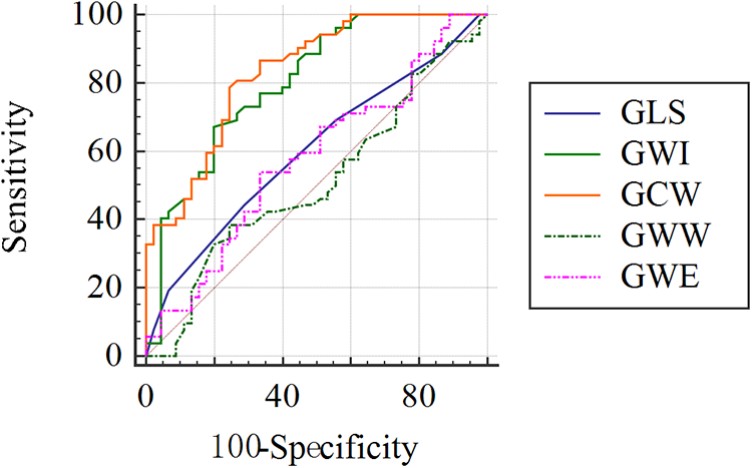
ROC curve analysis to evaluate the sensitivity and specificity of GLS, GWI, GCW, GWW, GWE for identifying impairment of LV systolic function in ANOCA patients with CMD.

We have summarized the data for echocardiography parameters for the two groups in Table 1. No discernible differences were evident between the CMD group and control group in LAVI, LVEDDI, LVEDVI, LVESVI, LVEF, mitral peak E velocity, mitral peak A velocity, E/A, e′ peak, E/e′, or S′ peak. The baseline APV was increased considerably in the CMD group compared with that in the control group [22.50 (range, 18.00–29.50) vs. 19.00 (16.50–23.50)]. Marked alterations did not occur in GLS, GWW, or GWE between the CMD group and control group. GWI and GCW were diminished strongly among CMD-group patients relative to controls [1913.50 (1786.75, 2121.75) vs. 2203.00 (2098.00, 2359.50) and 2265.73 ± 168.03 vs. 2497.27 ± 159.43, respectively; *P *< 0.001 for both] (Figure 2). GWI and GCW were moderately correlated with CFVR (*r* = 0.430, *P *< 0.001 and *r* = 0.538, *P *<* 0.001*, respectively) (Figure 3). In the multiple logistic regression analyses, GCW was identified as the only independent echocardiography parameter associated with CMD after adjusting for age and baseline APV [OR (95%*CI*) 1.009 (1.005–1.013); *P *<* *0.001] (Table 2). We employed ROC-curve analysis to determine the area under the curve (AUC), the sensitivity and specificity of MWIs and GLS for identifying LV function in ANONA patients with CMD (Figure 4). Based on ROC-curve analysis, GWI and GCW were superior to the other parameters tested for predicting CMD. The best threshold for identification of GWI for CMD was 2091 mmHg %, with a sensitivity of 67.3%, specificity of 80.8%, and AUC of 0.800. The best threshold for identification of GCW for CMD identification was 2,400 mmHg %, with a sensitivity of 78.8%, specificity of 75.6%, and AUC of 0.832.

**Table 2:** Univariate and multivariate logistic regression analysis for the prediction of CMD.

Variables	Univariate logistic regression analysis		Multivariate logistic regression analysis	
	*OR*-value(95%*CI*)	*P*-value	*OR*-value(95%*CI*)	*P*-value
Age, years	1.052 (1.004–1.103)	**0**.**034**	0.951 (0.908–0.996)	**0**.**035**
Baseline APV, (cm/s)	1.112 (1.001–1.223)	**0**.**028**	0.897 (0.817–0.984)	**0**.**022**
GWI (mmHg%)	0.998 (0.995–1.002)	0.342		
GCW (mmHg%)	0.993 (0.988–0.998)	**0**.**006**	1.009 (1.005–1.013)	**<0**.**001**

Comments are the same as Table 1. *P* < 0.05 is marked with bold type.

### 3.3. Intra- and inter-observer variability

GLS and all MWIs revealed excellent intra- and inter-observer relationships, with a minimum intraclass correlation coefficients (ICCs) value of 0.900 (Table 3).

**Table 3:** ICCs for intra- and interobserver variability for GLS and MW indices.

Variables	Intra-observer variability		Interobserver variability	
	ICCs	95% CI	ICCs	95% CI
GLS	0.934	0.860–0.968	0.900	0.791–0.953
GWI	0.926	0.844–0.966	0.915	0.820–0.959
GCW	0.936	0.866–0.970	0.925	0.842–0.964
GWW	0.950	0.894–0.976	0.947	0.889–0.975
GWE	0.951	0.897–0.977	0.949	0.892–0.976

ICCs, intraclass correlation coefficients. Comments are the same as Table 1.

## 4. Discussion

We revealed, for the first study time, the impairment of LV systolic function in ANOCA patients with CMD by MWIs. We demonstrated that: (1) GWI and GCW were strongly diminished among ANOCA patients suffering from CMD; (2) GWI and GCW were associated with CFVR; (3) GCW was the only independent predictor of CMD; (4) the best parameters from ROC curve to predict LV systolic function in ANOCA patients with CMD were GWI and GCW.

The primary characteristics of CMD are myocardial ischemia and hypoxia (21). CMD has a complex pathogenesis that is incompletely understood. The more accepted mechanism for CMD including endothelial dysfunction, dysfunction of the smooth muscle cells, and altered microvascular remodeling (22, 23). Endothelial dysfunction also leads to a reduction of the synthesis of nitric oxide. This action alters the balance between the generation and destruction of vessels, which results in microvascular rarefaction and decreased microvascular density. During the dysfunction of smooth muscle cells and impairment of microvascular remodeling, myogenic reactivity and tone are reduced, resulting in decreased myocardial perfusion (24). All these factors may lead to delivery of inadequate amounts of oxygen and nutrient to myocardial cells which, in turn, manifests as insufficient energy required for cardiac activity. CMD have impairment on the cardiac structure and function. The change in cardiac function is difficult to identify by conventional echocardiography in ANOCA patients with CMD. We examined the possibility of using MWIs to assess the minor changes of LV systolic function in these patients.

Studies have reported that, among all MWIs, GWI and GCW are the most robust indicators of ischemia in patients with CAD (25). In accordance with those studies, we revealed that GWI and GCW were decreased markedly in ANOCA patients with CMD. Myocardial work has been shown to have superior diagnostic value over traditional ultrasound parameters in several diseases. One study assessing myocardial function in terms of cardiometabolic demand reported good correlation between GWI and myocardial glucose metabolism measured by positron emission tomography (26). In ANOCA patients with CMD, repetitive ischemia and hypoxia in cardiomyocytes may have led to a decrease in utilization of myocardial energy and metabolic activity, which could have manifested as a decrease in GWI and GCW. Similarly, study on patients with obstructive sleep apnea syndrome, Jin et al. (27) also found a decreased of GWI and GCW.

We found that CFVR was positively correlated with GWI and GCW in patients with ANOCA. GCW was identified as the only independent echocardiography parameter associated with CMD after adjusting for age and baseline APV. In patients with CAD, CFVR can be used to predict cardiopulmonary fitness independent of resting systolic and diastolic function (28). In a prospective, multicenter, observational study by Cortigiani and colleagues, in 4,313 patients with known or suspected CAD who underwent SE for CFVR, the latter was a strong, independent indicator of mortality and had additional prognostic value compared with wall-motion analyses in patients with known or suspected CAD (29). Cardiac perfusion was also examined by analyses of CFVR: the reduction in CFVR might represent impairment of LV systolic and diastolic functions (30). Studies have shown a significant correlation between CFVR and LV diastolic function in patients with ANOCA (31). We demonstrated an association between coronary microvascular and LV systolic functions in ANOCA patients with CMD.

Our further analyses of GLS and MW parameters in patients with CMD revealed that the AUCs of GWI and GCW were significantly higher than those of GLS, GWW, and GWE. These data are similar to those from a study on patients with CAD by Guo and colleagues (25). They found GWI and GCW were the best parameters to detect LV segments with myocardial ischemia. For patients with CAD (especially single-vessel stenosis), regional MW measured by echocardiography has shown good diagnostic value for detecting significant myocardial ischemia compared with standard methods using fractional flow reserve. Myocardial ischemia and hypoxia are causes of myocardial dysfunction in ANOCA patients with CMD, which may be a direct factor leading to a decline in GWI and GCW. GWW represents myocardial lengthening during systole, along with any shortening during isovolumic relaxation. These contractions do not modulate LV ejection over time. With an increasing GWW, GWE is decreased. CMD is an early stage of disease, whereby there is no increase in GWW or decrease in GWE. Based on a PROMIS multinational study, the prevalence of CMD was relatively high among heart failure patients with preserved ejection fraction (HFpEF) (32). If CMD progresses to HFpEF, GWW, and GWE, the long-term implications are critical (33, 34).

In our study, the ROC results have found that GWI and GCW are moderate in predicting of CMD, but with low sensitivity and specificity. This may be related to the characteristics of our selected patients. We choose cases with coronary stenosis less than 30% and normal wall motion, which has little effect on the cardiac mechanical indices. This may lead to some overlap in MWIs between the CMD group and control group, so it will cause the decrease of sensitivity and specificity in the ROC curve We also found a moderate correlation between WMIs and CFVR, this may be related to the different measurement units of the two variables.

Several reports have suggested use of GLS for the diagnosis of myocardial ischemia, which may also be applicable as an adjunct diagnostic tool in the clinic (35, 36). Among patients with stable ischemic heart disease, GLS indicates the severity of CAD (37). However, the role of GLS in evaluating LV function among patients with ANOCA and CMD is controversial. More recently, Schroder J and colleagues suggested that GLS is diminished among patients with ANOCA, and this is linked intricately to CFVR (5). In contrast to their study, GLS in our study was similar between the control group and CMD group. This difference may have been because our population was different to that of their study: all of their patients were women, whereas ours were not. In a study by Michelsen and colleagues in patients with ANOCA and CMD, patients with angina pectoris typically displayed preserved GLS, with no correlation between baseline CFVR and GLS (38). The results of our study are consistent with the data of Michelsen and colleagues. In addition, the diagnostic precision of GLS assessment was likely affected by afterload at the time of data acquisition (39).

Given the evidence stated above, MWIs by LV PSL appears to be important for the diagnosis and prediction in patients with ANOCA and CMD. This new method for the quantitative evaluation of LV systolic function in patients with ANOCA and CMD is non-invasive and accurate.

## 5. Conclusions

In ANOCA patients with CMD, reduced LV systolic function may be associated with functional alterations within the coronary microcirculation. Our noninvasive LV PSL demonstrated incremental value for the identification of CMD in people suffering from ANOCA.

## 6. Study limitations

The present study had four main limitations. First, the study cohort was recruited from a single center. Second, the study was retrospective. Hence, in future investigations, we recommend a multicenter prospective investigation involving a large sample population to validate our findings. Second, CMR and positron emission tomography provide extensive information on the tissue profile and myocardial metabolism among patients with CMD. However, these data were not available for use during our study. Third, we assessed alterations in LV systolic function among patients with CMD only at rest: future research should involve examination of LV systolic function under stress.

## Data Availability

The raw data supporting the conclusions of this article will be made available by the authors, without undue reservation.
